# Broadening measures of success: results of a behavioral health translational research training program

**DOI:** 10.1186/s13012-017-0621-9

**Published:** 2017-07-24

**Authors:** Julie A. Baldwin, Heather J. Williamson, Emery R. Eaves, Bruce L. Levin, Donna L. Burton, Oliver T. Massey

**Affiliations:** 1 Northern Arizona University College of Health and Human Services, Center for Health Equity Research, 1395 S. Knoles Dr., #4065, ARD Building, Suite 140, Flagstaff, AZ 86011 USA; 2 Department of Occupational Therapy, Northern Arizona University College of Health and Human Services, 435 N 5th Street, Health Sciences Education Building, Phoenix, AZ 85004 USA; 3 Department of Child and Family Studies, University of South Florida College of Behavioral and Community Sciences, 13301 Bruce B. Downs Blvd., MHC 2321, Tampa, FL 33612 USA; 4 Department of Community & Family Health, University of South Florida College of Public Health, 13201 Bruce B. Downs Blvd., MDC 56, Tampa, FL 33612 USA

**Keywords:** Translational research, Implementation science, Behavioral health, Community engagement, Program evaluation

## Abstract

**Background:**

While some research training programs have considered the importance of mentoring in inspiring professionals to engage in translational research, most evaluations emphasize outcomes specific to academic productivity as primary measures of training program success. The impact of such training or mentoring programs on stakeholders and local community organizations engaged in translational research efforts has received little attention. The purpose of this evaluation is to explore outcomes other than traditional academic productivity in a translational research graduate certificate program designed to pair graduate students and behavioral health professionals in collaborative service-learning projects.

**Methods:**

Semi-structured qualitative interviews with scholars, community mentors, and academic mentors were conducted regarding a translational research program to identify programmatic impacts. Interviews were transcribed and coded by the research team to identify salient themes related to programmatic outcomes.

**Results:**

Results are framed using the Translational Research Impact Scale which is organized into three overarching domains of potential impact: (1) research-related impacts, (2) translational impacts, and (3) societal impacts. This evaluation demonstrates the program’s impact in all three domains of the TRIS evaluation framework. Graduate certificate participants (scholars) reported that gaining experience in applied behavioral health settings added useful skills and expertise to their present careers and increased their interest in pursuing translational research. Scholars also described benefits resulting from networks gained through participation in the program, including valuable ties between the university and community behavioral health organizations.

**Conclusions:**

This evaluation of the outcomes of a graduate certificate program providing training in translational research highlights the need for more community-oriented and practice-based measures of success. Encouraging practitioner involvement in translational research is vital to translate knowledge into practice and to enable practice-based needs to inform research and policy. A more flexible approach to measuring programmatic success in research training programs can help bridge the knowledge translation gap.

## Background

Translational research aims to improve individual and population health outcomes by bringing evidence-based knowledge into clinical and public health practice [1, 2]. Health service researchers and clinicians are increasingly expected to not only produce high-quality research but also facilitate sustained implementation of knowledge in practice [3]. Medical schools and research institutions face increasing pressure to improve researchers’ and clinicians’ competencies in both knowledge production and knowledge translation [4]. Evaluation of the success of training programs is therefore a priority.

Training in translational research has thus far demonstrated positive results. Targeting graduate students for mentoring has been shown to influence graduate students’ career development trajectories. Long-term mentoring also encourages support of mentees to continue to evolve their academic research careers [5]. Many evaluations, however, emphasize academic productivity (in the form of publications and grants) as a primary measure of success [6–8]. While the skills of the research team or their record of interdisciplinary collaboration have served as additional outcome measures in a few programs [9, 10], there is an increasing need to establish relevant competencies of trainees for evaluating success at all levels of the knowledge translation process. Whereas several frameworks for assessing success of translational research efforts exist, no consensus has been reached on how to best evaluate these programs [11–14].

In this paper, the authors present an evaluation of a National Institute on Drug Abuse (NIDA)-funded translational research training program, the Institute for Translational Research Education in Adolescent Drug Abuse (“the Institute”), aimed at engaging graduate-level scholars and behavioral health practitioners in substance abuse translational research. The Institute engages new researchers in combining substance abuse research with evidence-based approaches to implementation. Such a training program has potentially substantial public health impacts given the sizable gap between the demand for implementation of high-quality evidence-based programs and the number of researchers focusing in this area [15, 16].

Glasgow et al. [17] called for new dissemination and implementation research-practice collaborations that include those community members and local practitioners who will be involved in implementation. This involvement is key to embedding health care advances into existing workflows and achieving the sustainability that often eludes implementation science efforts [17]. Accordingly, Glasgow et al. [17] expanded the definition of translational research to a five-stage process involving overlapping and interrelated stages: 1) T0 is the identification of a health/behavioral health issue; 2) T1 is the development of interventions for the health/behavioral heath issue; 3) T2 is the identification of the interventions' impact on the health/behavioral health issue; 4) T3 is the evaluation of the implementation of these interventions in practice; and 5) T4 is the evaluation of the effectiveness of the intervention across several applied settings. The Institute’s focus is primarily in T3 translational research, which includes “investigations designed to increase uptake and implementation of evidence-based recommendations into practice” ([17], p1276).

One of the most challenging aspects of implementing evidence into practice is that implementation stakeholders (i.e. providers, organizations) rarely have the skills to interpret and implement new research into practice [18]. In this paper, the authors describe part of the Institute’s success in terms of building capacity for implementing evidence-based practices by training current and future practitioners and researchers to understand translational research and implementation within community settings. We advocate that traditional measures of success focusing on academic productivity (grants and publications) fail to capture the breadth of impact of a translational research institute intending to train community practitioners, future health professionals, and researchers in translational research.

### Institute overview

The central aim of the Institute is to develop innovative, applied research skills among behavioral health researchers and practitioners through a collaborative service-learning-focused approach. Institute scholars complete service-learning translational research and implementation science studies in collaboration with academic and community mentors and community behavioral health organization partners as part of the *Graduate Certificate in Translational Research in Adolescent Behavioral Health Program*. Implementation is not only relevant for academically based researchers but also has a pragmatic relevance for those who practice in the field. The intention of including both master’s and doctoral students in the Institute is to expose a broader range of professionals to the implications for translational research and implementation science in working agencies.

Service-learning studies are defined as research carried out as a service to the program. Scholars were expected to approach their projects based on what community mentors describe as the greatest need in their organization. The graduate certificate provided additional training for scholars, in most cases, outside of the requirements for their degree program. The program was designed as a graduate certificate to be available to enrolled graduate students and also available to community members seeking additional certification and/or knowledge for their current job. Additionally, making the program an official graduate certificate of the university created a sustainable program beyond grant funding.

Interested applicants applied to the Institute by submitting a personal statement, resume, two letters of recommendation, and official transcripts. Members of the Executive Committee then reviewed the applicants collectively and made final decisions regarding admission to the program. The Institute Executive Committee included the multiple principal investigators for the Institute and the project director who were responsible for the Institute’s design, implementation, and evaluation.

Two groups of mentors (academic and community) contributed to the delivery of the curriculum. The academic mentors provided support in research design and analysis, while the community mentors provided hands-on experience and real-world interpretations. Community agencies interested in participating in the Institute met with members of the Executive Committee to determine fit for the program, including the commitment to scholar project length and providing a community mentor at their site. Academic mentors were selected at the university based on areas of expertise that were applicable to the proposed research projects.

The graduate certificate program (15 credits) requires four continuous semesters, a total of 18 months, to complete. Scholars complete online coursework on adolescent behavioral health, translational research methods, and community-based participatory research, while also completing their in-person service-learning research project. During the first semester, scholars attend the *Annual Research and Policy Conference on Child, Adolescent and Young Adult Behavioral Health* where they participate in a networking event with community mentors and academic mentors. Scholars then rank preferences for projects, and the Executive Committee reviews scholar preferences and mentor expertise and places the scholars and mentors into their respective research teams. During the last semester of the program, graduating scholars also attend the conference (cited above) to present findings from their applied research studies. For additional information about the Institute, see the website, http://www.usf.edu/cbcs/cfs/itre/index.aspx [19, 20].

As part of the triadic mentoring relationship, scholars have the opportunity to gain experience in improving community readiness, uptake, and successful implementation of new programs and practices. Academic mentors provide experience in methodologies of data collection, research and evaluation methods, community consultation, and problem solving, while community partners provide the practical application possibilities and pitfalls of accomplishing such changes.

Measures for gauging the success of the program beyond academic productivity include real-world implementation and community outcomes. These measures are considered a step toward a more comprehensive understanding of the impact of translational research training on both scholars and community behavioral health organizations.

The purpose of this evaluation is to explore outcomes of the Institute beyond academic productivity in order to highlight areas of impact and suggest potential next steps in the field of translational research. The Translational Research Impact Scale (TRIS), developed by Dembe et al. [14], provides a framework for evaluating long-term and community-oriented outcomes of the Institute. The TRIS distinguishes three domains of potential impact: (1) research-related impacts, (2) translational impacts, and (3) societal impacts. Expansion to translational and societal impacts lends a broader perspective to evaluation, moving beyond academic productivity to consider implementation and impact as key outcomes of translational research training. For a full list of potential impact indicators in each sub-domain of the TRIS, refer to Dembe et al. [14].

## Methods

### Participants and data collection

#### Scholars

The Institute engages in ongoing process and outcomes evaluation in its efforts to inform practitioners and future researchers about the field of translational research and implementation science. The current study reports evaluation measures completed to date with the first two cohorts of scholars enrolled in the graduate certificate program. Evaluation is mainly focused on cohort #1 scholars, for whom information about career trajectory and longer term impact has already been collected. Cohort #1 included a Masters of Social Work student, six Masters of Public Health students, four employees of community agencies, two doctoral students in public health, and one doctoral student in criminology. Insights from cohort #2 scholars (similar in background), as well as academic and community mentors, are also included.

Cohort #1 scholars (completed Institute participation in May 2014) were interviewed 1 year after completion of the Institute graduate certificate program. Questions focused on long-term outcomes of participation in the Institute, including current career, current interests and engagement in translational research, dissemination and research activities, and other themes of interest. Cohort #2 scholars (completed Institute participation in May 2015) were interviewed about their experiences in the Institute and plans for future research, career aspirations, and beliefs about how the Institute influenced their careers and professional aspirations overall.

#### Community mentors

For each cohort, Institute scholars were matched with a community partner organization focused on behavioral health in order to complete the translational research and implementation science study. For cohort #1, five community behavioral health organizations agreed to host a team of scholars and provide an on-site community mentor to work directly with scholars on the conceptualization and implementation of the translational research study. The on-site community mentor provided oversight on the nature and scope of the project, while also assisting the scholars with ongoing data collection and analysis activities. For cohort #2, all but one of the original five agencies continued their involvement with the Institute.

#### Academic mentors

In addition to being paired with a community mentor, scholars were also paired with academic mentors, forming a triadic mentoring relationship. Academic mentors were tasked with providing consultation and oversight of research methodology including qualitative and quantitative approaches for data collection and analysis. In particular, academic mentors provided oversight on methodology in order to ensure the service-learning translational research studies best matched the needs of the behavioral health organizations. Academic mentors were paired with teams of scholars whose proposed research studies matched at least one of their areas of research expertise.

### Data analysis

The purpose of this evaluation was to assess longer term impacts of the Institute on scholars as well as from the perspectives of academic mentors and community partners. This evaluation was approved by the University of South Florida Institutional Review Board and participants provided verbal consent to participate. Semi-structured qualitative interviews were audio recorded and transcribed verbatim. A member of the Executive Committee trained the interviewers. Interviewers conducted practice interviews with Institute staff and met to discuss issues that arose in order to align strategies prior to conducting the interviews.

Sample size was limited by the total number of people participating in the Institute program. In contrast to quantitative sampling in which large numbers of participants are required to achieve significance, relatively small numbers, as few as twelve in some instances, of participants are typically needed in qualitative research to achieve what is referred to as “thematic saturation” [21–24]. Thematic saturation is achieved when additional interviews no longer uncover unique themes in participants’ comments. Because of the narrow scope of the questions explored in this evaluation, we believe that the total number of interviews (*n* = 33) was sufficient for the purposes of this evaluation.

All interview transcripts were coded manually using both inductive and deductive approaches [23–26] for themes related to outcomes and impacts of interest for the Institute evaluation. Pre-determined areas of interest used to identify themes included: (1) *career aspirations*, (2) *current career*, (3) *dissemination activities*, (4) *education activities/aspirations*, (5) *grant-writing activities/aspirations*, and (6) *influence of the Institute on Scholars*’ *lives overall*. The importance of *networks gained through Institute participation* was added as an emergent theme of central interest to scholars and community mentors. Two coders met multiple times to discuss coding and establish reliability. This reliability procedure was intended to critically reflect on disagreements and improve analytical consistency, not to measure percentage of agreement [27, 28]. Qualitative analysis of codes was performed by an independent analyst not affiliated with the Institute with expertise in qualitative analysis methods and theoretical analysis [29–31]. Involving a third party in analysis helped to reduce bias and bring a fresh perspective to assess Institute outcomes.

After initial coding and analysis, the Translational Research Impact Scale (TRIS) [14] was used to frame the potential impact of translational research efforts of the Institute. This evaluation demonstrates the Institute’s impact in all three domains of the TRIS evaluation framework (see Dembe et al. [14]).

## Results

Institute scholars were recruited from health- and social-services-related graduate academic disciplines, programs, and fields, such as criminal justice, education, nursing, psychology, public health, behavioral health, rehabilitation and mental health counseling, and social work. Of the fifteen scholars in cohort #1, twelve agreed to be interviewed for this evaluation. Of the twelve scholars in cohort #2, seven agreed to be interviewed for this evaluation.

Community mentors were full-time employees at local or regional child and adolescent behavioral health service agencies. We use behavioral health service agencies to refer to organizations that provided mental health, substance use, and/or educational programs, in this case specific to children and adolescents. Three of the agencies provided direct behavioral health services. One agency disseminates and evaluates an evidence-based curriculum focused on preventing substance abuse. The final agency was the social work services division of a local county school system. All five community mentors representing participating community behavioral health service agencies participated in the 2014 evaluation. Three out of the four community mentors participated in the 2015 evaluation.

Of the academic mentors, two were psychologists with backgrounds working with community and school-based mental health intervention programs. One academic mentor had a background in mental health policy and social/health psychology and extensive experience working with community behavioral health agencies on evaluation projects. Another academic mentor was a sociologist with expertise in behavioral health statistics. All six of the academic mentors involved with the cohort #1 scholars participated in interviews during the 2014 evaluation. Three of the four academic mentors involved with the cohort #2 scholars participated in interviews during the 2015 evaluation.

Using the domains of the TRIS framework [14] (research-related impacts, translational impacts, and societal impacts) as a guide, the authors describe the range of ways participation in the Institute impacted both scholars and community mentors. Overall, scholars and community mentors reported increased interest in translational research, implementation science, and substance abuse research as a result of participating in a research project as part of the Institute service-learning component, as well as development of valuable professional networks and ongoing collaborative partnerships. The impacts in each of the TRIS domains follow and quotes from scholars, community mentors, and academic mentors illustrate their respective impact in specific areas of the TRIS domains.

### Research-related impacts

Domain 1, *research related impacts*, is the most extensive domain in the TRIS. Research-related impacts include areas such as (1) identification of research needs, (2) development of innovative methods, (3) improvement of research conduct and/or management strategies, (4) improvements in analysis and synthesis of results, (5) increases in the number of grant submissions and publications among translational researchers, (6) new, marketable discoveries, and (7) successes in research dissemination to appropriate audiences. Table 1 contains illustrative quotes from participants to show research-related impacts resulting from Institute-applied research projects.

**Table 1:** Illustrative quotations to demonstrate areas of research-related impacts

▪ Research networks and collaborations formed ▪ Translational researchers recruited and trained	*I also formed a lot of relationships through the team that I worked with for my Institute project. So, that influenced a lot of where I ended up going, it impacted the topic of my master’s thesis project. Really, it led to the current job I’m doing now. So, in providing technical assistance, a lot of my area that I contribute to on our larger team has to do with implementation and evidence-based practice. So I pull a lot from that in my current work (Cohort #1 Scholar)* *I had never considered going into research when I started my Master’s program but as a result of being involved in the Institute and being involved in that process, that’s the trajectory of my career now. (Cohort #1 Scholar)* *I was pleasantly surprised that these [Community Mentors], although they are practice people, they were very much interested in research and they were very much interested in collaboration. I was pleasantly surprised at the level of the knowledge and their willingness to collaborate. (Academic Mentor)*
▪ New collaborative, translational research projects completed ▪ Researchers involved in problem solving and creating novel ways of addressing barriers	*The opportunity of the Institute was actually to focus and narrow down my research interest to find my true passion towards adolescent health in general and a reason in particular which has been included in my dissertation proposal. I think the Institute was a milestone in order to get there, to narrow down that ideal, learning everything in the big picture. (Cohort #1 Scholar)* *We had two service learning projects that actually all our staff—I supervise social workers—identified. So I took the projects to the scholars or to the Institute. My position was to kind of explain what was involved. So I provided the leadership for that and then I would say after that the scholars embraced it, added to it, worked on it, both came out with products that we are able to utilize. (Community Mentor)* *It’s, I believe, in a lot of ways expanded my scope of research. …One of the things I did learn in the Institute that helped is doing more interdisciplinary research where a proven fact that’s in another field might be of use to something that I’m doing in my field. So that’s certainly, I’m hoping, adds up to a new way of looking at things and problems. (Cohort #1 Scholar)*
▪ Grants and manuscripts submitted or underway	*I’m still very close with the community partner that I did the research with and we are still working on the manuscript … They’ve been quite an asset for me, I hope that I’ve been able to help them a little bit as well. (Cohort #1 Scholar)* *I wrote my special projects about my experience in the Institute and I have a publication pending for that. I did a poster presentation at the Society for Prevention Research based off our findings from my Institute involvement. (Cohort #1 Scholar)* *The enhancement part came in for writing. I’ve actually learned how to put together a manuscript for this particular population. I know how to write, but knowing how to write about a project that I did in a team specifically for community research, that was pretty cool. (Cohort #2 Scholar)*
▪ Translational research is disseminated to the local and national researchers and community organizations	*[The Institute] gave me a lot of resources in assessing whether or not a program is strong for the community in working with mental health. That is a big tool for me to see if the agency that I’m working with is impacting the community around it in a positive way. (Cohort #1 Scholar)* *I feel like I have so many more resources and places to look for things that really matter for our end of implementation in the field. (Cohort #1 Scholar)*

A range of new research networks and collaborations were formed as a result of collaborative mentoring and community-based participatory research studies conducted as part of the Institute graduate certificate program. Community mentors, representing five different community behavioral health organizations collaborated with Institute scholars on community-based applied research studies. Although some individuals had prior relationships with the Institute’s faculty, community mentors and academic mentors reported improved collaborative relationships as a result of the Institute service-learning component [32].

The triangular research mentoring relationship, including scholars, academic mentors, and community mentors, reportedly influenced scholars’ success and helped them develop and refine competencies in public health and translational research [19]. One year after participation in the Institute, scholars reported that networks gained through experience with the Institute were instrumental in obtaining their present positions and both scholars and community mentors reported those networks strengthened their commitment to pursuing community-engaged work initiatives.

To date, four cohorts of scholars have created and implemented applied research studies in collaboration with community mentors. The service-learning studies that were completed have positively impacted the operations of participating community organizations [20]. The translational research studies completed by Institute scholars in collaboration with community mentors ranged from exploring the integration of behavioral health and primary care services (traditionally delivered separately in the United States) to identifying factors and mechanisms for adapting evidence-based practices in schools. Scholars and community mentors reported heightened awareness of the need for practical application of evidence-based research.

In the context of translational research, individual mentoring can be difficult and thus not conducive to collaborative training. The Institute developed a collaborative mentoring approach to address this challenge and provided broader training to scholars as well as offering beneficial service-learning research to community behavioral health organizations [19]. Emphasis on the “real-world” or actual community agency experience provided by the Institute was a key theme in scholars’ descriptions of the overall impact of the Institute on their lives and career aspirations. Scholars reported that as a result of participation in the Institute, they had a better appreciation for the importance of research in applied community settings, as well as the need for evidence-based understanding prior to implementation and intervention.

Inclusion of community mentors and practice-oriented scholars led to widespread diffusion of understanding of the techniques involved in conducting and implementing translational research. Scholars reported giving presentations to local policymakers and community organizations. Those scholars working for health organizations also reported bringing techniques learned from participating in the Institute into their work and educating their colleagues on the value of evaluation.

Five scholars from cohort #1 reported presenting study results at national conferences. Several manuscripts are in preparation, authored by scholars and mentors from cohorts #1 and #2. Thus far, one manuscript has been published by an Institute scholar team [33]. Scholars cited the short timeline of participation in the Institute, the need for more incentives to publish, and inexperience in academic writing as barriers to manuscript preparation, submission, and subsequent publication.

One scholar noted that over-emphasis on academic productivity can frustrate practice-oriented scholars and de-emphasize outcomes that are of importance to community organizations such as programmatic development or sharing results with other providers.

*Sometimes we get really wrapped up in formal research, in publishing and getting our names on things. That kind of left a bitter taste about that, not specifically from the Institute just like in general through my master’s program. I think maybe that’s why I’ve been not as involved or like eager to go to conferences or eager to be in the American Public Health Association or anything, just because I feel like we end up getting kind of really lost in that. I’m much more of like a one-on-one kind of person, like hands-on on the ground type work. (Cohort #1 Scholar)*

To balance the different outcomes valued by academia versus community organizations, one key outcome for all projects included in the Institute was a presentation at a national conference held in Tampa, FL (*Annual Research and Policy Conference on Child, Adolescent, and Young Adult Behavioral Health*). Disseminating project results at this conference created an opportunity to speak to both academic- and community-based audiences.

### Translational impacts

Domain 2, *translational impacts*, includes potential impact in areas such as (1) incorporation of discoveries from bench science into human or animal studies, (2) incorporation of clinical trial results into clinical or practical guidelines, (3) recruitment and preparation of new translational researchers, and (4) improvement in evidence-based health care service and delivery, patient outcomes, and positive health behaviors, as a result of research translation. Table 2 provides illustrative quotes from Institute participants regarding translational impacts.

**Table 2:** Illustrative quotations to demonstrate translational impacts

▪ Scholars trained in translational research methods are engaged in collaborative partnership with various community health organizations ▪ People with the skills to conduct translational research and implementation are involved with local organizational programming	*The impact on the staff I think was that I think it highlighted for them a little bit more of where the emphasis should be and, again, outside of just what’s covered by the evidence-based models because right now there is the more evidence-based prevention model that really deals with prescription meds as a specific issue. So I think it clarified a bit more what things we can do in the short term to help to actually manage that. (Cohort #1 Scholar/Community Mentor)* *Just like the ability to conduct research, kind of like the technical aspects of it as well. That’s not easy and not everyone knows how to do it or knows who to contact or knows the pieces of how research is implemented. So, the Institute really helped me in understanding that. (Cohort #1 Scholar)*
▪ Emphasis on real-world application of evidence is fostered	*I just think that it’s a way of living, it’s a way of being, it’s a way of doing things, whether it’s in your place of employment or it’s interacting with people. My eyes are much more open to many of these social issues, not just in criminology and crime, but many of the social issues that are plaguing our society now. I have just like a much better understanding of that information and where to go and get good quality research on that. Definitely, it’s shaped the way that I think of the world. (Cohort #1 Scholar)* *It’s made me think about working with the community more. I’m a community girl. I come from the community and it’s made me realize like, “Hey, you know what, if it’s going to be relevant at all to life, it needs to come from the community.” I don’t want my research projects to come from my brain and from the ivory tower anymore. I want them to be a communication with people who actually could benefit. (Cohort #2 Scholar)* *Anywhere we can find meaningful data to understand how our programs are used and how we can help share what we think are best practices. I just think it continues to be a good benefit for both us and for the university. (Community Mentor)*

The Institute’s inclusion of graduate students and working professionals from a range of health- and social-services-focused disciplines resulted in a diverse cohort of professionals trained in translational research methodology not only from the university but also from community agencies. Whereas transdisciplinary research across university departments is important in furthering research and translation, members of community-based organizations involved in implementation also benefit from training in translational research and implementation science methods. As earlier Institute-focused publications describe, the processual development of the Institute, including a community agency network and a translational research training program, have contributed toward the goal of increasing the number of people working in health care who have been trained to understand the importance of evidence translation and research [19, 20, 32].

### Societal impacts

Domain 3, *social impacts*, includes potential impact areas such as (1) strengthening and refining of health-related policies and procedures, (2) improvements in community health, empowerment, and economic conditions to reduce health disparities, and (3) development of new community-based participatory programs and partnerships geared toward effective and meaningful implementation. For illustrative quotes related to societal impacts resulting from Institute projects, see Table 3.

**Table 3:** Illustrative quotations to demonstrate impacts related to local community and society

▪ Community-based participatory programs are developed and implemented ▪ Findings are shared with local policy-makers and organizations	*We presented the results of the project that we worked on to their leadership council. Then, we did some recommendations on how to improve their implementation of evidence-based programs. We also presented back to again the substance abuse coalition in [the] County that we worked with a little bit through this project. We presented our findings to them as well. (Cohort #1 Scholar)*
▪ A network of researchers and community members was created ▪ Practice-focused researchers were trained to use evidence-based strategies in their organizations	*[The Institute] gave me a lot of resources in assessing whether or not a program is strong for the community in working with mental health. That is a big tool for me to see if the agency that I’m working is impacting the community around it in a positive way. (Cohort #1 Scholar)* *The community-based participatory research or just the way that you engaged with experts and who they are … I think that has broadened not only my knowledge base, but just my approach to life and to research and to hopefully what I can do in the future. (Cohort #1 Scholar)*
▪ Community organizations awareness of the need for evidence-based practices and for improved evaluation increased	*I think, more than anything, it’s just given us insight that there is an ongoing need to change, that being stagnant and relying on what we’ve always done is not healthy and certainly doesn’t work for us or the people that we’re trying to serve. So there is an intention to continue to be part of any effort to look at how the research and the application of the knowledge gained through the research gets done, much more than I think we ever have had in the past. (Cohort #1 Scholar/Community Mentor)* *After the project we are now reassessing additional training that may need to be included to ensure implementers have the proper guidance and support. (Community Mentor)*

Service-learning applied research studies completed as part of the Institute’s graduate certificate and training program were collaboratively designed and implemented by scholars and community mentors and academic mentors. The emphasis on mutual benefit was designed to ensure the sustainability of community-based behavioral health programs and partnerships*.* While most scholars did not continue to collaborate with the community mentors after graduating from the program, the community mentors continued partnerships with the university through ongoing service-learning projects with new cohorts of Institute scholars. Rather than attempts to create new behavioral health programs, the Institute’s training program was designed to train new researchers as well as make meaningful contributions to the function and applicability of existing behavioral health programs. Scholars have presented study outcomes and insights to local policymakers and community behavioral health organizations. Such presentations contribute to participatory decision-making and policy development. Community mentors described impacts to their organizations in terms of understanding the value of applied research and collaboration with the University, as well as of the need for adaptation and innovation of their ongoing operations.

Examples of outcomes from service-learning translational research projects completed as part of the Institute’s graduate certificate program include the following: (1) development of a training for providers focused on appropriate mechanisms to adapt a school-based evidence-based practice curriculum; (2) implementation of a professional development training program focused on mental health counseling; and (3) identifying facilitators and barriers to integrating primary care and behavioral health services.

The Institute’s emphasis on community-university partnerships with a community-based participatory research foundation was designed to broadly impact child and adolescent behavioral health issues [32]. Community behavioral health organizations involved in the Institute reported benefits as a result of collaboration with scholars and improved methods for serving their targeted at-risk populations.

At the time of the evaluation, cohort #1 scholars were employed in a range of behavioral health organizations and university departments. Current careers included the following: technical assistants on health focused grant projects; reporting and analytics for a health care organization; senior qualitative analyst for a health care organization; administrator for a substance-abuse-focused organization; curriculum development and implementation specialist for a substance abuse outreach organization; manager for a children’s special needs organization; and research fellow for a federal healthcare organization.

As noted above, one of the most challenging aspects of implementing evidence into practice is that implementation stakeholders (i.e., providers, organizations) rarely have the skills to interpret and implement new research into practice [18]. Part of the Institute’s societal impact is in providing training, particularly in T3, to people employed in various community organizations.

## Discussion

Implementing a multi-component, applied service-learning research experience, in addition to education or perhaps curriculum-based translational research, holds the potential to expand the public health and behavioral health workforce in needed areas [34, 35]. To monitor the success of such programs, however, knowledge about translational research and implementation science must be considered along with evidence of productivity [36]. Evaluation of translational research education training must therefore include measures of success beyond conventional academic metrics of peer-reviewed publications. In this paper, several areas of impact beyond academic productivity are highlighted. Dimensions such as community engagement, network building, and practical application of evidence are areas of potential impact in translational research and implementation science; however, such effects have not been emphasized or measured in translational research education training [13].

Fortunately, there are resources available to encourage translation of research into practice. The National Collaborating Centre for Methods and Tools [37] has developed a series of resources for practitioners, including education and self-assessments, to encourage their uptake and use of evidence-based practices. Approaches to evaluating the success of translational research education training require sufficient flexibility to accommodate individuals and individual institutions, while still retaining enough rigor to demonstrate that a program is meeting established objectives and competencies [2]. Another potential outcome of translational research education could be utilizing evidence to inform policy making. The SUpporting POlicy Relevant Reviews and Trials (SUPPORT) Project provides a series of resources targeting policy-makers to encourage their use of evidence-informed decision-making in policy development [38].

Training and mentoring new researchers in implementation science and in translational research are increasingly emphasized in health equity research, as evidenced by the many current programs in existence for training both students and faculty members [1, 6, 8, 10, 39–41]. To facilitate innovation in translational research in clinical and services research, researchers from various disciplines must be willing to engage in collaboration beyond the defined margins of their discipline [9]. Although most education training is focused on early career research faculty, Wooten et al. [4] suggest that engaging graduate-level scholars in translational research may be the key to inspiring a new cohort of investigators in this field. A critical hurdle in translating knowledge to practice is the gap that exists between academic publication goals and the policy-oriented interests of public health and behavioral health directors and service agency administrators. There is a need to establish effective iterative loops in translation between researchers and those involved in implementation in order to address shared goals [15].

### Limitations

This paper is limited by the self-reported nature of the qualitative data collected. Qualitative investigation is well-suited, however, to providing in-depth information about participants’ experiences. For the purpose of this evaluation, qualitative interviews were intended to elicit information about stakeholder experiences in the Institute. Although this paper does not provide a comprehensive evaluation of the Institute, these findings about Institute scholar experiences and the need for alternative measures of success will likely be of interest to similar current and future educational training programs in translational research [8, 39–41]. Findings related to the relevance of translational research training to members of community behavioral health organizations also point to the importance of training in these skills beyond academia.

## Conclusions

Over the course of this evaluation, the authors identified several drawbacks in emphasizing academic measures of productivity rather than community outcomes in evaluating translational research training programs. Common measures, such as numbers of grant applications and awards, are less revealing of success when applied to practice-oriented graduate and professional degree programs and community organizations. In this paper, the use of the TRIS domains to identify areas of potential impact suggests ways of broadening measures of success in the context of a translational research educational training program designed for graduate students, community partners, and academic mentors. This triadic, applied approach to translational research training has potential to broaden the reach of translational research in several ways. After participating in training programs, researchers are better prepared to work in interdisciplinary environments and to successfully implement evidence-based programs in applied community practice settings. Further, training not only graduate students but also members of stakeholder organizations can facilitate improved communication and understanding and lead to stronger community/university partnerships.

## Abbreviations

NIDA: National Institute on Drug Abuse
TRIS: Translation Research Impact Scale
